# The Apolipoprotein E (*APOE*) Gene Appears Functionally Monomorphic in Chimpanzees (*Pan troglodytes*)

**DOI:** 10.1371/journal.pone.0047760

**Published:** 2012-10-24

**Authors:** Annick M. McIntosh, Calvin Bennett, Dara Dickson, Stephanie F. Anestis, David P. Watts, Timothy H. Webster, M. Babette Fontenot, Brenda J. Bradley

**Affiliations:** 1 Department of Anthropology, Yale University, New Haven, Connecticut, United States of America; 2 Division of Behavioral Sciences, New Iberia Research Center, University of Louisiana at Lafayette, Lafayette, Louisiana, United States of America; Erasmus University Medical Center, The Netherlands

## Abstract

**Background:**

The human apolipoprotein E (*APOE*) gene is polymorphic, with three primary alleles (E2, E3, E4) that differ at two key non-synonymous sites. These alleles are functionally different in how they bind to lipoproteins, and this genetic variation is associated with phenotypic variation for several medical traits, including cholesterol levels, cardiovascular health, Alzheimer’s disease risk, and longevity. The relative frequencies of these alleles vary across human populations, and the evolution and maintenance of this diversity is much debated. Previous studies comparing human and chimpanzee *APOE* sequences found that the chimpanzee sequence is most similar to the human E4 allele, although the resulting chimpanzee protein might function like the protein coded for by the human E3 allele. However, these studies have used sequence data from a single chimpanzee and do not consider whether chimpanzees, like humans, show intra-specific and subspecific variation at this locus.

**Methodology and Principal Findings:**

To examine potential intraspecific variation, we sequenced the *APOE* gene of 32 chimpanzees. This sample included 20 captive individuals representing the western subspecies (*P. troglodytes verus*) and 12 wild individuals representing the eastern subspecies (*P. t. schweinfurthii*). Variation in our resulting sequences was limited to one non-coding, intronic SNP, which showed fixed differences between the two subspecies. We also compared *APOE* sequences for all available ape genera and fossil hominins. The bonobo APOE protein is identical to that of the chimpanzee, and the Denisovan *APOE* exhibits all four human-specific, non-synonymous changes and appears functionally similar to the human E4 allele.

**Conclusions:**

We found no coding variation within and between chimpanzee populations, suggesting that the maintenance of functionally diverse *APOE* polymorphisms is a unique feature of human evolution.

## Introduction

**Figure 1:**
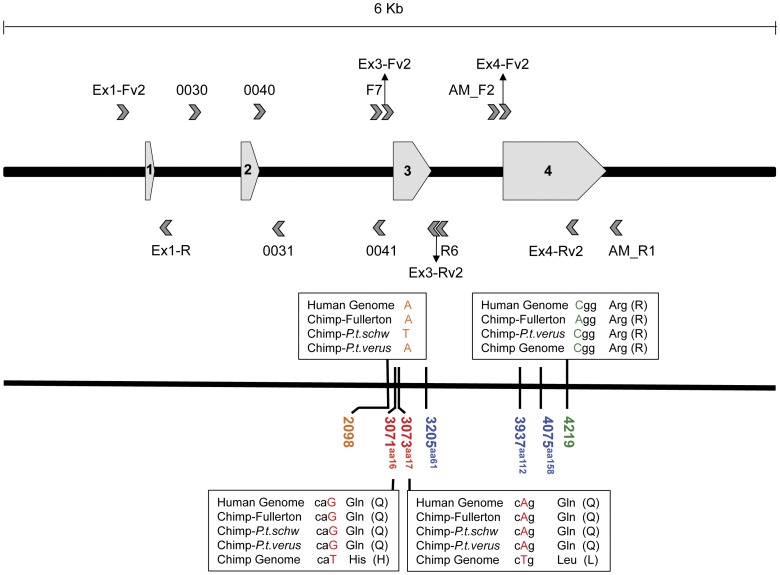
A schematic of the *APOE* gene. Structure and nucleotide position numbers follow Fullerton *et al*. [27] and Ensembl (ENSG00000130203). The location of primers used in this study are given above (forward primers) and below (reverse primers) the labeled exons. See Table S1 for primer and PCR-cycling information. An intronic SNP differentiating the two chimpanzee populations is highlighted in orange (position 2098*). SNP locations in red (3071 and 3073) represent putative *APOE* non-synonymous changes based on the chimpanzee genome assembly (Pan_troglodytes-2.1.4). Positions in blue (3205, 3937 and 4075) correspond to the amino acids (61, 112 and 158, respectively) that define the three human *APOE* alleles (E2, E3, E4). Position 4219^†^ (in green) represents the single, synonymous difference between the *P. t. verus* sequences generated in this study and that of Fullerton *et al.* (2000) [27]. *corresponds to Ensembl coordinates 19∶45411002 for the human genome and 19∶50097633 for the chimpanzee genome. **^†^**corresponds to Ensembl coordinates 19∶45412223 for the human genome.

*APOE*, which codes for apolipoprotein E (CCDS ID: 12647), is among the best-studied genes in the human genome [1], [2] and is a frequently-cited case study in discussions of evolutionary medicine [3]–[8]. *APOE* is polymorphic in humans, with three primary alleles: E2, E3, and E4 [9], [10]. These three alleles differ at two key non-synonymous sites, resulting in amino acid differences at positions 112 and 158 (Table 1, Figure 1, Figure S1). This genetic variation has been causatively linked with variation in total serum cholesterol, LDL cholesterol levels, and general cardiovascular health [11], [12]. *APOE* variation has also been associated with aspects of cognition and brain development [12]–[14], susceptibility to infectious disease [1], and longevity [15], [16]. Notably, the E4 variant is considered a potential risk factor for medical conditions ranging from heart disease [17]–[20] to Alzheimer’s disease [21]–[23].

Human APOE differs from that of other mammals at amino acid residue 61, which is arginine in humans but threonine in non-human mammals [24] (Figure 1). The interaction between residues 61 and 112 influences protein structure and lipoprotein binding, with the human E4 binding preferentially to low-density lipoproteins, while the E2 and E3 variants bind preferentially to high-density lipoproteins [24], [25]. Although the APOE of non-human mammals and the human E4 allele both have arginine residues at 112 and 158, studies of transgenic mice indicate that the threonine at position 61 in the APOE of non-human mammals results in binding preferences that are functionally similar to human E3 [26]. Thus, although the human E4 is thought to be the ancestral allele for humans, the ancestral primate APOE protein likely had lipid-binding properties most similar to E3.

E3 is the most common allele in humans, but frequencies vary across human populations, ranging from ≤50% in populations in Burkina Faso and Brazil to ≥90% in certain tribal groups [27], [28]. Population genetic analyses date the increase and spread of the E3 variant to about 200,000 years ago [27], by which time organized hunting, and potentially cooking [29], were part of the human behavioral repertoire [30]. Noting that lipoproteins can play an important role in dietary function, Finch and colleagues [4], [5], [31] have suggested that the E3 allele might be a “meat-adaptive” variant associated with increased animal consumption during human evolution. Others [3] argue that the E3 amino acid changes (at 112 and 158) were more likely compensatory mutations minimizing the deleterious mutation (at residue 61), which was likely fixed by chance earlier in the human lineage. In any case, sequence [27] and simulation [15] studies suggest that the increased frequency of E2 and E3 in human evolution was likely driven by selection.

Thus, understanding the function and diversity of APOE in humans requires an evolutionary context, and our understanding of APOE is often framed in terms of human-chimpanzee comparisons [3], [4], [5], [27], [31]. Researchers [5] have urged caution, however, as these comparisons are generally based on APOE data from the single chimpanzee (of the western subspecies, *Pan troglodytes verus*), sequenced by Fullerton et al. [27]. The APOE sequence from the chimpanzee genome also generally represents a single western chimpanzee, Clint [32]. Although a restriction enzyme study [33] verified that codon positions 112 and 158 were monomorphic in 24 captive chimpanzees (presumably all representing the western subspecies), it is unclear whether chimpanzees show functional variation elsewhere in the protein. Moreover, since chimpanzee populations differ in their degree of hunting and meat eating [34]–[37], the extent of population and subspecific variation at this locus is also of interest.

To examine this, we sequenced the *APOE* coding region for 32 individuals from two sample populations of chimpanzees: captive western chimpanzees (*Pan troglodytes verus*) and wild eastern chimpanzees (*Pan troglodytes schweinfurthii*). We found no exonic sequence variation in these populations, confirming that the human *APOE* functional polymorphisms are recently derived and human-specific.

## Results

We found no exonic sequence variation among individuals within or between chimpanzee populations. Because DNA samples from the captive, western chimpanzee population (*P. t. verus)* were obtained from blood, while DNA samples from the wild, eastern chimpanzees (*P. t. schweinfurthii*) were obtained from lower-quality fecal material (see Materials and Methods), the wild population was sequenced at a subset of *APOE* target regions. We initially focused our efforts on exons 3 and 4 (Figure 1), where virtually all *APOE* sequence variation is found in humans. A total of 32 individuals (20 *P. t. verus*, 12 *P. t. schweinfurthii*) and 20 individuals (20 *P. t. verus*) were sequenced for exons 3 and 4, respectively, but no variation was observed within or across chimpanzee subspecies. Moreover, all sequence trace-files indicated homozygous sequences; no heterozygous sites were observed. A minimum of 65 chromosomes are necessary to detect a 5% polymorphism with 95% power, while 40 chromosomes are sufficient to detect a 5% polymorphism with 80% power [38]. Thus, if chimpanzees were polymorphic at these loci, we would likely have detected any variation present with our sample sizes of 64 chromosomes for exon 3 and 40 chromosomes for exon 4.

We also sequenced several *P. t. verus* at exon 1 (n = 4) and exon 2 (n = 10), and found no variation. These short (exon 1∶143 bp; exon 2∶66 bp) exons likely have minimal influence on the resulting protein structure; in humans, exon 1 is untranslated and only a portion of exon 2 is translated (ENST00000252486). Humans also show little variation at these exons, as the 1000 Genomes browser [39] (accessible via Ensembl (ENSG00000130203)) indicates only one SNP in human exon 1 (rs72654467) and no variation in exon 2. Similarly, Fullerton et al. [27] found no variation at these exons across human populations.

We sequenced 65% of the *APOE* intronic regions, including portions of all three introns (Figure 1, Table S1), and found no within-population variation. However, one single nucleotide polymorphism (SNP) was found between exons 2 and 3 (position 2098, Figure 1), which showed fixed differences between the two subspecies. At this site, *P. t. schweinfurthii* differs from *P. t. verus* and humans by the replacement of adenine (A) with thymine (T). Such fixed differences between the western and eastern subspecies are not uncommon and reflect the genetic structure of chimpanzee populations [40], [41]. This SNP site has a low GERP (Genomic Evolutionary Rate Profiling based on 35 eutherian mammals) conservation score of −2.91, suggesting that this region has not been subject to strong purifying selection [42]. In any case, our data indicate that nucleotide diversity at APOE is low in chimpanzees (within subspecies≈0; across subspecies = 0.037%), especially in contrast to other coding regions of the genome [43].

Notably, our sequences differ at one synonymous site in exon 4 (position 4219, Figure 1) from the previously published chimpanzee *APOE* sequence of Fullerton et al. [27] (Genbank accession: AF261280), and at two adjacent non-synonymous sites in exon 3 (positions 3071 and 3073 in Figure 1) from the *APOE* sequences in the chimpanzee genome assemblies available in the Ensembl and UCSC browsers. Whether these are real polymorphisms or sequencing errors is unclear. Since the chimpanzee genome sequence was largely derived from a single individual representing the western subspecies, *P. t. verus* [32], it is possible, although unlikely, that this individual possessed rare mutations at this gene. These SNPs would result in Gln34His and Gln35Leu substitutions (Figure 1), which have BLOSUM matrix scores [44] of 0 and −2, respectively, indicating that such substitutions are not especially common.

The translated amino acid sequence of the chimpanzee genome as currently presented in Ensembl (ENSPTRG00000011127) differs from the chimpanzee sequences generated in this study and from the human genome at a string of six amino acids coded at the beginning of exon 2 (Figure S1). This translation indicates a complete replacement of six codons (Ensembl chimpanzee translation: ATGGGGGCGGGGCTTGCT coding for MGAGLA). We compared the chimpanzee genome *APOE* sequence and the APOE translation given for the chimpanzee genome in the UCSC browser (UCSC coordinates chr19∶50095613–50099088), and found that this difference is due to an Ensembl translation of a partial intronic sequence.

**Figure 2:**
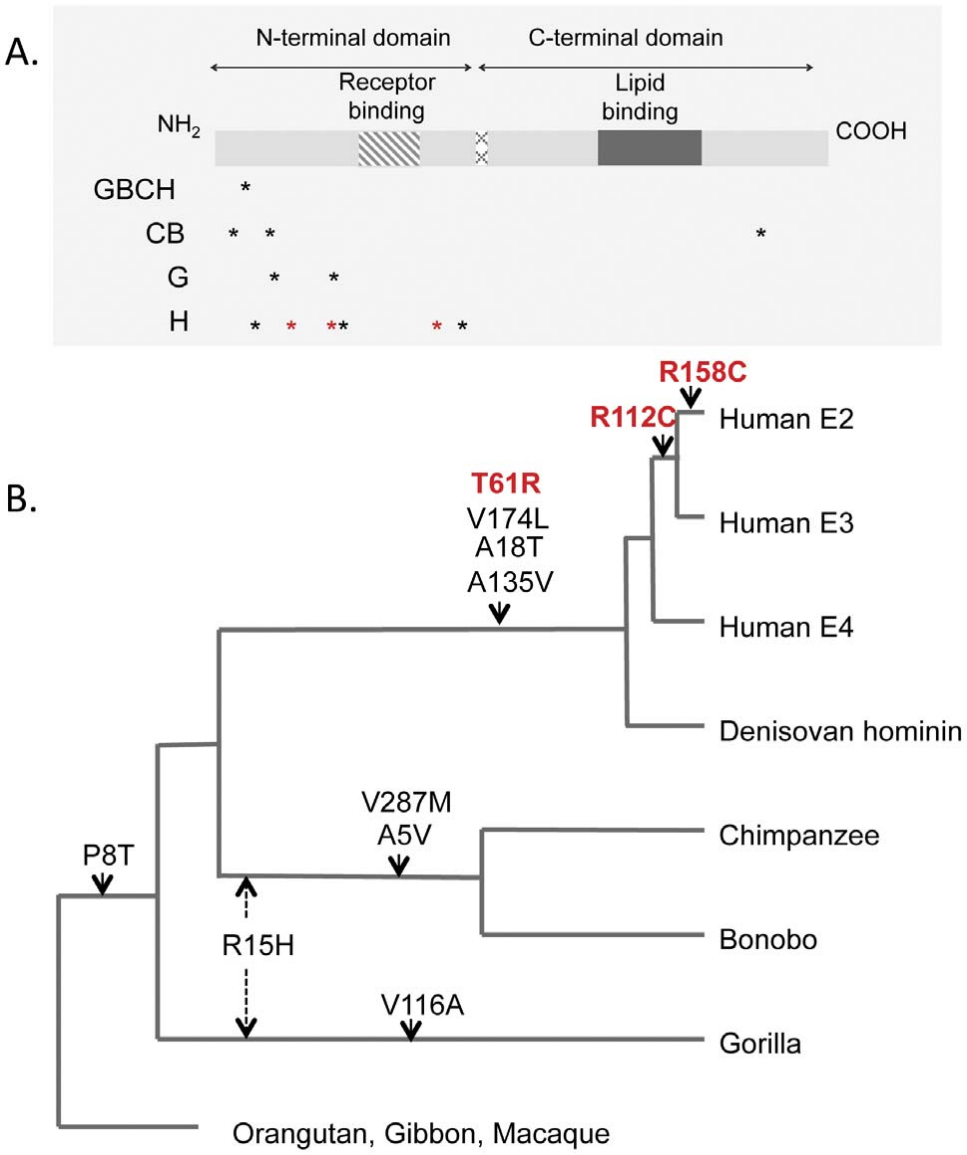
Lineage-specific mutations mapped onto a schematic of the APOE protein (A) and primate phylogeny (B). Protein structure is modeled after Bu 2009 [65], and tree topology represents known evolutionary relationships based on genome-wide data [46]. Human mutations [66] at key residues 61, 112 and 158 are in red. Including residue 61, the human APOE protein has four fixed, *Homo*-specific, non-synonymous mutations, all of which seem to be shared with the Denisovan hominin (inferred from reads mapped to the human genome at http://www.genome.ucsc.edu). The chimpanzee APOE protein is monomorphic within and between subspecies, and is identical to the bonobo APOE protein. Mutation R15H (dotted arrow) is shared by gorillas, chimpanzees and bonobos likely as a result of incomplete lineage sorting rather than independent evolution [46].

We also examined *APOE* sequence data from other recently generated primate genomes of interest, including the bonobo (*Pan paniscus*) [45], the western gorilla (*Gorilla gorilla*) [46], the Neanderthal (*Homo neanderthalensis*) [47], and the Denisovan hominin [48], [49]. Translated protein sequences were aligned (Figure S1), and lineage-specific mutations identified (Figure 2) using the orangutan and gibbon as out-groups (see Materials and Methods).

**Table 1:** Variation at key *APOE* functional sites in *Homo* and *Pan*.

	Gene location* (amino acid)		
	3205 (61)	3937 (112)	4075 (158)
UCSC human coordinates	chr19∶50,103,049	chr19∶50,103,781	chr19∶50,103,919
Ensembl human coordinates	19∶45411209	19∶45411941	19∶45412079
dbSNP IDs		rs429358	rs7412
Human APOE2	AGG: Arg	**T**GC: Cys	**T**GC: Cys
Human APOE3	AGG: Arg	**T**GC: Cys	**C**GC: Arg
Human APOE4	AGG: Arg	**C**GC: Arg	**C**GC: Arg
Chimpanzee	A**C**G: Thr	**C**GC: Arg	**C**GC: Arg
Bonobo	A**C**G: Thr	**C**GC: Arg	**C**GC: Arg
Denisovan	AGG: Arg	**C**GC: Arg	**C**GC: Arg
Neanderthal	A**AA**: Lys**	Unknown	unknown

* Gene nucleotide positions following notation of Fullerton et al. 2000 [27], and as given in Figure 1.

** Based on two reads, one each from two fossil specimens: Vi33.25 and Vi33.26.

The translated APOE sequence of the bonobo genome is identical to that of the chimpanzee sequence generated in this study (Figure S1). Only a partial protein sequence was available for gorilla (Figure S1), but we identified two derived amino acid differences in the gorilla lineage, one of which is shared with *Pan* but not *Homo*, likely due to incomplete lineage sorting [46], [50]. Notably, terminal branch *d*_N_/*d*_S_ values [51] are low and do not differ markedly among human (0.240), chimpanzee (0.288) and orangutan (0.254).

Reads from the Denisovan hominin suggest an APOE protein similar to the human APOE4 [3], [49] (Table 1), with arginine at key functional amino acid sites 61, 112 and 158 (Table 1). The mapped reads from the Denisovan also indicate matches with the three other human-specific mutations at residues 18, 135, and 174 (Figure 2B).

No sequence reads from the Neanderthal genome correspond to regions coding for amino acid sites 112 and 158. The Neanderthal genome has two reads, from two different Neanderthal specimens, that cover residue 61. These give the same codon AAA, which if correct, would code for lysine, rather than the AGG coding for arginine typical of human APOE (Table 1). However, these ostensible G to A transitions (Table 1), which occur at the 3′ end of the reads, are quite possibly errors induced by guanine deamination and nucleotide misincorporation during high-throughput sequencing [52]–[54]. Nonetheless, given that residue 61 distinguishes the human APOE from other mammals, and that mutations at this amino acid are known to modify lipid-binding properties [26], possible variation in Neanderthals at this site should be further examined.

## Discussion

This study provides the first empirical demonstration that the APOE amino acid sequence is monomorphic in chimpanzees. Unlike humans, chimpanzees show little genetic variation and no protein variation at this locus. This is striking, as chimpanzees generally exhibit higher levels of genetic diversity, even at the sub-specific level, compared to humans [55]. In addition, our analysis reveals that the chimpanzee APOE protein sequence is identical to the APOE sequence translated from the bonobo genome, although the extent of intra-specific variation among bonobos is unclear, as the bonobo genome was generated from a single individual [45].

The APOE sequence translated from the current chimpanzee genome assembly (panTro4) differs from our 33 identical amino acid sequences (the 32 individuals sequenced in this study plus the one sequenced by Fullerton et al. [27]) at two adjacent non-synonymous sites in exon 3 (Figure 1). However, these differences may represent chimpanzee genome sequencing errors, and should be viewed with caution. Indeed, we also found that the translated chimpanzee APOE protein given in the Ensembl genome browser includes, what appears to be, a mistakenly translated partial intron (Figure S1).

Plotting the lineage-specific, non-synonymous mutations along the protein (Figure 2A) and the ape phylogeny (Figure 2B) highlights several features of APOE evolution. The receptor-binding (N-terminal) domain and the lipid-binding (C-terminal) domain are completely conserved (see also Figure S1) among African apes. Given that the C- and N-terminal domain interaction of human APOE4 is thought to play a role in neuropathology (e.g. Alzheimer’s risk) [26], [56], it is notable that all but one of the non-synonymous mutations fall in the N-terminal domain of the protein. This includes the mutation thought to cause the domain interaction (T61R) and the compensatory mutation proposed to prevent it (R112C) [3]. The single mutation in the C-terminal domain is one of only three amino acid changes found in chimpanzees (and bonobos) but not humans (Figure 2B).

The one Denisovan hominin sequenced to date has all four of the fixed, human-specific APOE substitutions (A18T, T61R, A135V, and V174L; Figure 2B) and matches the ancestral human E4 allele (112R and 158R). This might not be surprising, given the high frequency of the E4 allele in Melanesians [10] and the purported similarities among Denisovan and New Guinea/Bougainville genomes [48].

Although there is variation in the amount of hunting and meat-eating among chimpanzee populations [34]–[37] and between chimpanzees and bonobos [57], this protein is highly conserved across *Pan*. Thus, selective pressures associated with these differences in meat consumption are not associated with genotypic or protein differences in APOE. This does not undermine the hypothesized role of APOE in the evolution of meat eating in humans, though, as the *Pan* APOE protein is predicted to be functionally similar to the human E3 (“meat adaptive” [4], [5], [31]) allele. Notably, ischemic heart disease seems to be rare in at least some wild [58] and captive [59] populations of chimpanzees. Moreover, chimpanzees consume significantly less meat than human hunter-gathers [60].

While the chimpanzee populations in this study do not display functional APOE protein variation, we cannot exclude the possibility that chimpanzees exhibit regulatory or intronic variation that influences APOE expression and/or function. Transgenic models and physiological studies of chimpanzee- and gorilla-specific variants of APOE are needed to understand the functional adaptation of this key protein among apes, including humans. Such comparative analyses provide the context for evolutionary interpretations of APOE, and its role in human uniqueness and disease risk.

## Materials and Methods

### Ethics Statement

Sources of DNA included both blood and fecal samples. Fecal samples were collected non-invasively from chimpanzees in the Kibale National Park, Uganda (Ngogo community). Permission to conduct this research was granted by the Uganda Wildlife Authority and the Ugandan National Council of Science and Technology. Blood samples were collected serendipitously by veterinarians at the New Iberia Research Center when chimpanzees were anesthetized for other reasons (standard health checks). All animals at NIRC are housed and handled in strict accordance with good animal practice as defined by the University of Louisiana at Lafayette Institutional Animal Care and Use Committee, following the US Public Health Service Policy on Humane Care and Use of Laboratory Animals, and all animal work was approved by this committee. Protocol approval numbers for Institutional Animal Care and Use Committees are: University of Louisiana at Lafayette IACUC#2010-8707-053; Yale University IACUC#2010-11378.

### Tissue Samples and DNA Extractions

Blood samples from twenty unrelated individuals representing the western chimpanzee subspecies (*Pan troglodytes verus*) were collected during routine health checks at the New Iberian Research Center (NIRC Lafayette, Louisiana). Fecal samples were collected from fifteen unrelated wild individuals in the Ngogo community, Kibale National Park, Uganda [61]. These individuals represent the eastern chimpanzee subspecies (*Pan troglodytes schweinfurthii*). We extracted DNA using the QIAGEN DNeasy Blood & Tissue Kit and the QIAGEN Stool Kit following the manufacturer’s protocols with the following modification: fecal samples were incubated at 24**°**C for 24–48 hours prior to applying to spin columns. Extraction procedures were automated via a QIAcube (QIAGEN). DNA concentrations were estimated using either a Nanodrop 2000 (Thermo-Fisher Scientific) spectrophotometer (DNA from blood) or a quantitative PCR assay (most DNA extracts from fecal samples [62]).

### APOE Amplification and Sequencing

We used a combination of previously published and novel primer sequences (Figure 1, Table S1). To construct primers, we imported and aligned *APOE* sequences for multiple primate species via GenBank (www.ncbi.nlm.nih.gov/genbank), Ensembl (ensembl.org), and UCSC (genome.ucsc.edu) genome browsers (Figure S1). We designed new primers based on conserved flanking regions surrounding each exon, and we used the Fullerton et al. primers [27] spanning the intronic region between exons 2–3. Primer attributes were assessed using NetPrimer (Premier Biosoft) and then synthesized by Eurofins MWG Operon. All primer sequences are available in Table S1.

After optimizing cycling conditions for new primer pairs, we amplified each segment via PCR. For high quality, blood-derived DNA (20 *P. t. verus* samples), we prepared the following 25 µl PCR reaction as follows: 1.25 Units QIAGEN HotStarTaq Master Mix, 400 nM each primer, 1.25 µl (5%) DMSO, and 2 µl template DNA (10–50 µg). For lower quality fecal-extracted DNA (12 *P. t. schweinfurthii*), we incorporated the following reagents into a 20 µl PCR reaction: 2 Units 10× buffer, 1.75 mM MgCl_2_, 6 µg BSA, 200 nM each primer, 200 µM dNTPs, 0.25 Units SuperTaq (Invitrogen), and 3 µl template DNA (>100 pg). PCR reactions were carried out in Geostorm thermocyclers under the following conditions: after an initial denaturation at 94**°**C for 5 min, reactions underwent 35 cycles of 94**°**C for 30 sec, 54–65**°**C (see Table S1) for 30 sec, and 72**°**C for 45 sec, followed by a final extension at 72**°**C for 7 min. Negative controls were included in all reactions.

PCR products were visualized on a 1.8% agarose gel, containing GelRed (Biotium) along with DNA ladders (100 bp, New England BioLabs). We then purified and sequenced successful amplifications on an Applied Biosystems 3730×l DNA Genetic Analyzer at the Yale DNA Analysis Facility. All products were sequenced, and confirmed, in both directions. Resulting sequence trace files were imported and aligned using Genesifter, Sequencher 4.9 (Gene Codes), and eBioX software. All chromatograms with Phred quality scores below 20 were automatically discarded, and trace file quality was always confirmed visually. The generated chimpanzee *APOE* sequences were submitted to GenBank (accession: JX826621). Pairwise differences were calculated using Arlequin [63].

### APOE Sequence Retrieval for Other Primates and Fossil Hominins

We utilized APOE sequences from the chimpanzee genome assembly available in the UCSC (Pan_troglodytes-2.1.3 or panTro3) and Ensembl (Pan_troglodytes-2.1.4 or panTro4) genome browsers for further intraspecific comparison.

For further analyses, we obtained *APOE* sequence data from other recently generated primate genomes of interest, including the bonobo (*Pan paniscus*; NCBI: ERP000601&2) [45], and western gorilla (*Gorilla gorilla*, Ensembl: gorGor3) [46]. Sequences were translated and aligned (Figure S1) using eBioX, and lineage-specific mutations were identified (Figure 2) using the orangutan (*Pongo pygmaeus*, Ensembl: PPYG2) and white-cheeked gibbon (*Nomascus leucogenys*, Ensembl: Nleu1.0) as out-groups. We retrieved terminal branch *d*_N_/*d*_S_ values, calculated using orthology with the gibbon via Ensembl’s implementation of codeml in PAML [51]. This encompassed only those species annotated in Ensembl, which does not yet include the bonobo genome [45].

We also examined sequence data from the Neanderthal [47] and Denisovan [48] genomes, mapped to the human *APOE* sequence (hg19), via the UCSC browser (http://genome.ucsc.edu/Neandertal/). These data are small reads generated from six (Neanderthal) and one (Denisovan) fossil specimens, and given the inherent problems of sequencing ancient DNA (e.g. nucleotide misincorporations due to deamination [64]), the sequences must be interpreted with caution. We specifically focused on sites of functionally interesting polymorphisms, and we included only reads with the high base quality and alignment quality scores.

## Supporting Information

Figure S1 **Aligned primate APOE protein sequences.** Human allele E3 is shown. Fullerton *et al.* refers to the chimpanzee sequence generated in reference #27. Other sequences were retrieved and translated from the respective primate genomes. The translation of the chimpanzee APOE amino acid sequence given in the Ensembl genome browser (“Chimp, Ensembl trans.”, ENSPTRT00000061867) differs from our translation (“Chimp genome”) and that found in the UCSC browser. Note that this represents the full APOE protein precursor, which translates as 317 amino acids. *APOE* sequences generated from mRNA are often truncated and begin at residue 18 [66], thus the key amino acid sites 61, 112, and 158 correspond to sites 79, 130 and 176 (boxed), respectively, in the full protein. The receptor-binding domain (light gray shading) and the lipid-binding domain (dark grey binding) are completely conserved across these primate species, and the majority of the fixed, species-specific mutations fall in the N-terminal domain (see also Figure 2 in main text).
(TIF)

Table S1 Primer sequences, product sizes and annealing temperatures for amplification protocols used in this study. See Figure 1 in main text for relative locations of primer pairs.
(XLSX)
