# Supplementary figures and images for: The Apolipoprotein E (APOE) Gene Appears Functionally Monomorphic in Chimpanzees (Pan troglodytes)
Source: PLoS One. 2012 Oct 24;7(10):e47760. doi: 10.1371/journal.pone.0047760 (PMC3480407; doi:10.1371/journal.pone.0047760)

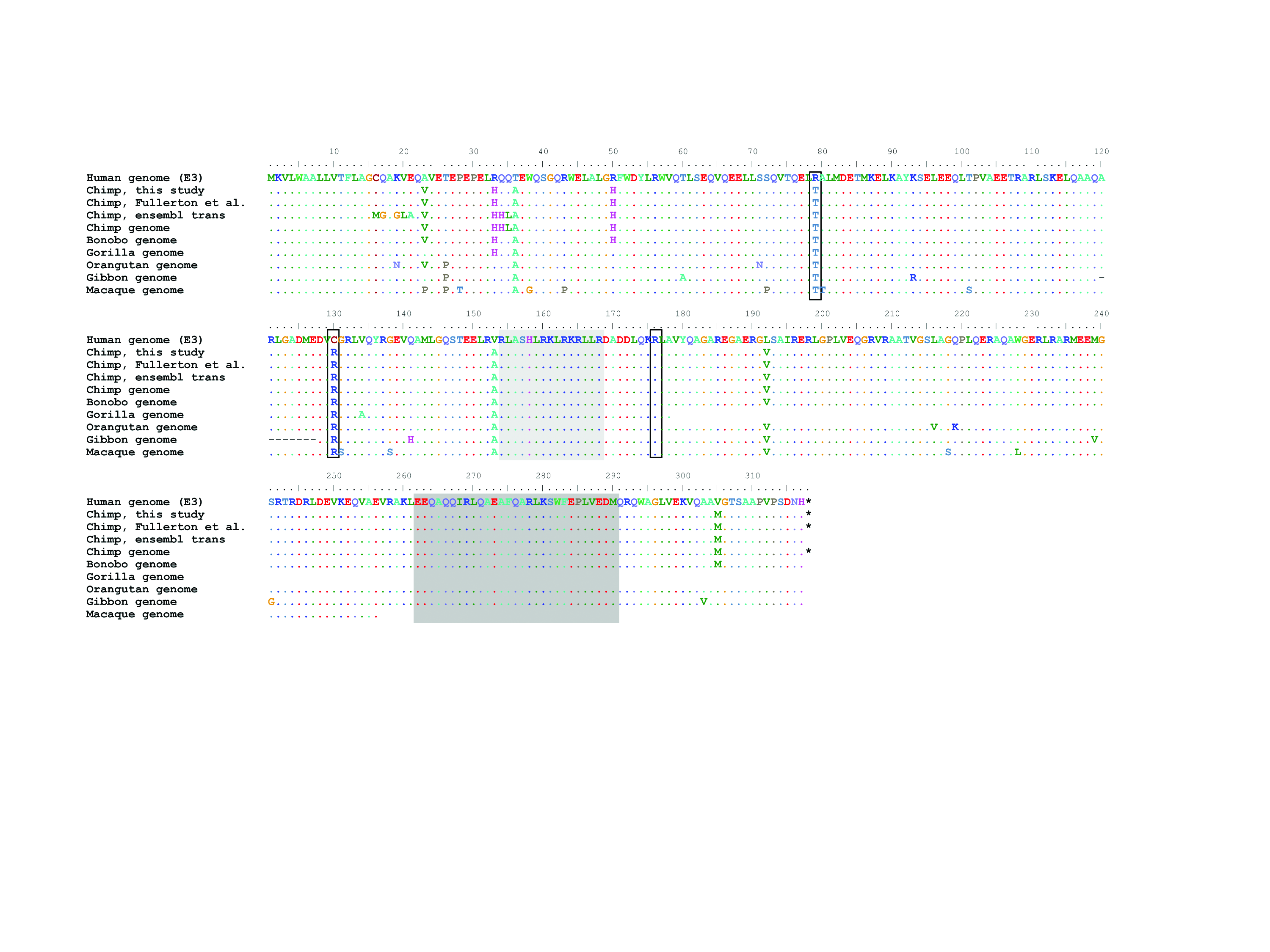

Supplement: Figure S1 — Aligned primate APOE protein sequences. Human allele E3 is shown. Fullerton et al. refers to the chimpanzee sequence generated in reference #27. Other sequences were retrieved and translated from the respective primate genomes. The translation of the chimpanzee APOE amino acid sequence given in the Ensembl genome browser (“Chimp, Ensembl trans.”, ENSPTRT00000061867) differs from our translation (“Chimp genome”) and that found in the UCSC browser. Note that this represents the full APOE protein precursor, which translates as 317 amino acids. APOE sequences generated from mRNA are often truncated and begin at residue 18 [66], thus the key amino acid sites 61, 112, and 158 correspond to sites 79, 130 and 176 (boxed), respectively, in the full protein. The receptor-binding domain (light gray shading) and the lipid-binding domain (dark grey binding) are completely conserved across these primate species, and the majority of the fixed, species-specific mutations fall in the N-terminal domain (see also Figure 2 in main text). (TIF) [file pone.0047760.s001.tif]
